# Dynamic Facial Expression of Emotion and Observer Inference

**DOI:** 10.3389/fpsyg.2019.00508

**Published:** 2019-03-19

**Authors:** Klaus R. Scherer, Heiner Ellgring, Anja Dieckmann, Matthias Unfried, Marcello Mortillaro

**Affiliations:** ^1^Department of Psychology and Swiss Center for Affective Sciences, University of Geneva, Geneva, Switzerland; ^2^Department of Psychology, University of Würzburg, Würzburg, Germany; ^3^GfK Verein, Nuremberg, Germany

**Keywords:** dynamic facial emotion expression, emotion recognition, emotion enactment, affect bursts, appraisal theory of emotion expression

## Abstract

Research on facial emotion expression has mostly focused on emotion recognition, assuming that a small number of discrete emotions is elicited and expressed via prototypical facial muscle configurations as captured in still photographs. These are expected to be recognized by observers, presumably via template matching. In contrast, appraisal theories of emotion propose a more dynamic approach, suggesting that specific elements of facial expressions are directly produced by the result of certain appraisals and predicting the facial patterns to be expected for certain appraisal configurations. This approach has recently been extended to emotion perception, claiming that observers first infer individual appraisals and only then make categorical emotion judgments based on the estimated appraisal patterns, using inference rules. Here, we report two related studies to empirically investigate the facial action unit configurations that are used by actors to convey specific emotions in short affect bursts and to examine to what extent observers can infer a person's emotions from the predicted facial expression configurations. The results show that (1) professional actors use many of the predicted facial action unit patterns to enact systematically specified appraisal outcomes in a realistic scenario setting, and (2) naïve observers infer the respective emotions based on highly similar facial movement configurations with a degree of accuracy comparable to earlier research findings. Based on estimates of underlying appraisal criteria for the different emotions we conclude that the patterns of facial action units identified in this research correspond largely to prior predictions and encourage further research on appraisal-driven expression and inference.

## Introduction

A comprehensive review of past studies on facial, vocal, gestural, and multimodal emotion expression (Scherer et al., 2011) suggests three major conclusions: (1) emotion expression and emotion perception, which constitute the emotion communication process, are rarely studied in combination, (2) historically, most studies on facial expression have relied on photos of facial expressions rather than on dynamic expression sequences (with some exceptions, e.g., Krumhuber et al., 2017), and (3) the research focus was mainly on emotion recognition, particularly recognition accuracy, rather than on the production of facial expressions and the analysis of the cues used by observers to infer the underlying emotions.

There are some notable exceptions to these general trends. Hess and Kleck (1994) studied the extent to which judges rating videos of encoders' spontaneously elicited and posed emotions could identify the cues that determined their impression of spontaneity and deliberateness of the facial expressions shown. They used the Facial Action Coding System (FACS; Ekman and Friesen, 1978) to identify eye movements and the presence of action unit (AU) 6, crow's feet wrinkles, expected to differentiate spontaneous and deliberate smiles (Ekman and Friesen, 1982; Ekman et al., 1988). They found that AU6 was indeed reported as an important cue used to infer spontaneity even though it did not objectively differentiate the eliciting conditions. The authors concluded that judges overgeneralized this cue as they also used it for disgust expressions. In general, the results confirmed the importance of dynamic cues for the inference of spontaneity or deliberateness of an expression. Recent work strongly confirms the important role of dynamic cues for the judging of elicited vs. posed expressions (e.g., Namba et al., 2018; Zloteanu et al., 2018).

Scherer and Ceschi (2000) examined the inference of genuine vs. polite expressions of emotional states in a large-scale field study in a major airport. They asked 110 airline passengers who had just reported their luggage lost at the baggage claim counter, to rate their emotional state (subjective feeling criterion). The agents who had processed the claims were asked to rate the passengers' emotional state. Excerpts of the videotaped interaction for 40 passengers were rated for the underlying emotional state by judges based on (a) verbal and non-verbal cues or (b) non-verbal cues only. In addition, the video clips were objectively coded using the Facial Action Coding System (FACS; Ekman and Friesen, 1978). The results showed that “felt,” but not “false” smiles [as defined by Ekman and Friesen (1982)] correlated strongly positively with a “in good humor” scale in agent ratings and both types of judges' ratings, but only weakly so with self-ratings. The video material collected by Scherer and Ceschi in this field study was used by Hyniewska et al. (2018) to study the emotion antecedent appraisals (see Scherer, 2001) and the resulting emotions of the voyagers claiming lost baggage inferred by judges on the basis of the facial expressions. The videos were annotated with the FACS system and stepwise regression was used to identify the AUs predicting specific inferences. The profiles of regression equations showed AUs both consistent and inconsistent with those found in published theoretical proposals. The authors conclude that the results suggest: (1) the decoding of emotions and appraisals in facial expressions is implemented by the perception of sets of AUs, and (2) the profiles of such AU sets could be different from previous theories.

What remains to be studied in order to better understand the underlying dynamic process and the *detailed mechanisms* involved in emotion expression *and* inference is *the nature of the morphological cues in relation to the different emotions expressed and the exact nature of the inferences of emotion categories from these cues*. In this article, we argue that the process of emotion communication and the underlying mechanisms can only be fully understood when the process of emotional expression is studied in conjunction with emotion perception and inference (decoding) based on a detailed examination of the relevant morphological cues—the facial muscle action patterns involved. Specifically, we suggest using a Brunswikian lens model approach (Brunswik, 1956) to allow a comprehensive dynamic analysis of the process of facial emotion communication. In particular, such model and its quantitative testing can provide an important impetus for future research on the dynamics of emotional expression by providing a theoretically adequate framework that allows hypothesis testing and accumulation of results (Bänziger et al., 2015).

**Figure 1:**
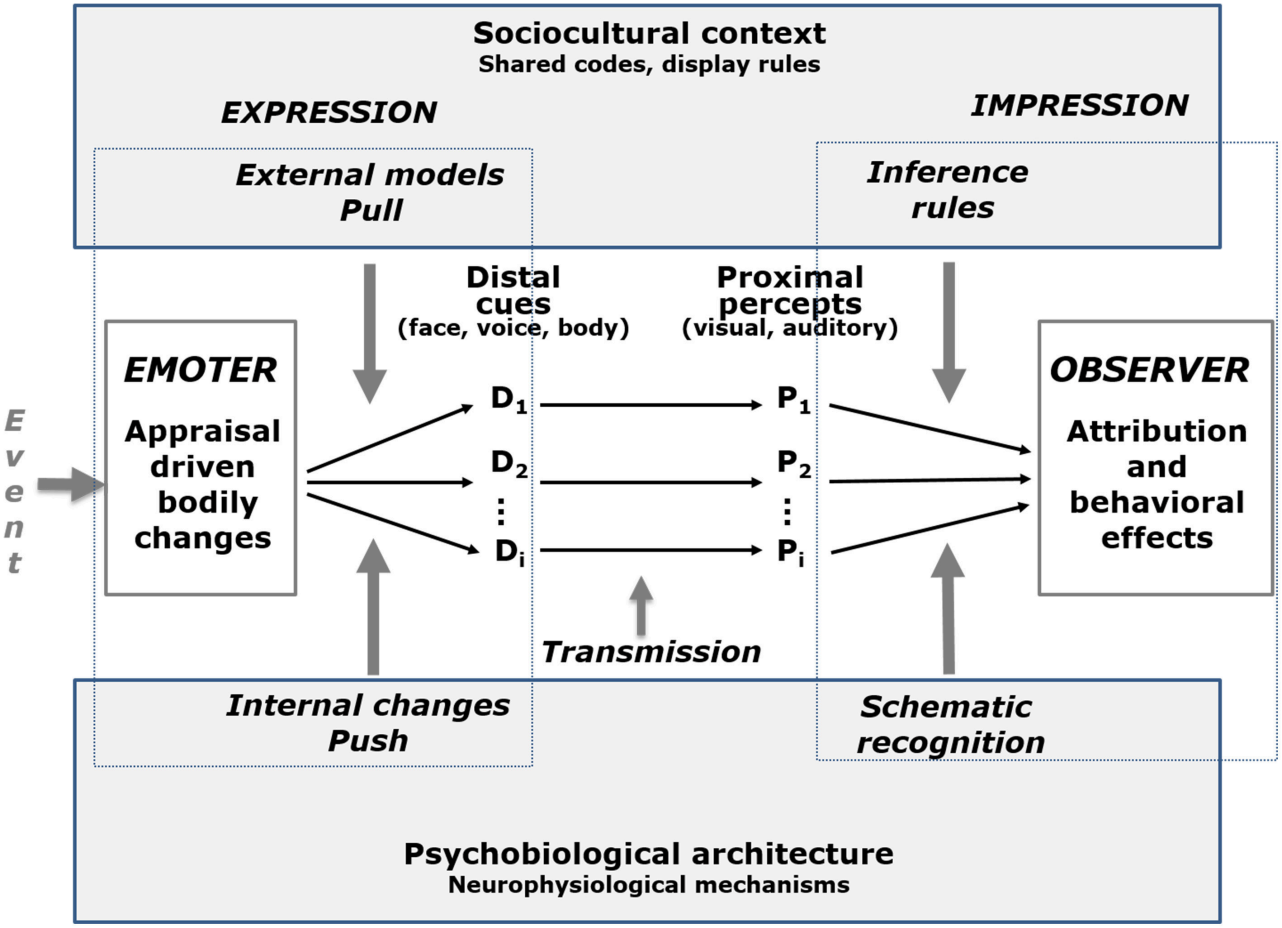
The tripartite emotion expression and perception (TEEP) model (based on Brunswik's lens mode). The terms “Push” and “Pull” refer to the internal and the external determinants of the emotional expression, respectively, distinguished in the lower and upper parts of the figure. ANS, autonomous nervous system; SNS, somatic nervous system. Adapted from Scherer (2013a, Figure 5.5). Pull effects refer to an expression that is shaped according to an external model (e.g., a social convention), Push effects refer to internal physiological changes that determine the nature of the expressive cues.

Scherer (2013a) has formalized an extension of the lens model as a tripartite emotion expression and perception (TEEP) model (see Figure 1), in which the communication process is represented by four elements and three phases. The internal state of the sender (e.g., the emotion experienced) is encoded via distal cues (measured by objective, quantitative analysis); the listener perceives the vocal utterance, the facial expression and other non-verbal behavior and extracts a number of proximal cues (measured by subjective ratings obtained from naive observers), and, finally, some of these proximal cues are used by the listener to infer the internal state of the sender based on schematic recognition or explicit inference rules (measured by naive observers asked to recognize the underlying emotion). In Brunswikian terminology, the first step in this process is termed the externalization of the internal emotional state, the second step the transmission of the behavioral information and the forming of a perceptual representation of the physical non-verbal signal, and the third and last step the inferential utilization and the emergence of an emotional attribution.

Despite its recent rebirth and growing popularity, the lens model paradigm has rarely been used to study the expression and perception of emotion in voice, face, and body (with one notable exception, Laukka et al., 2016). Scherer et al. (2011) reiterated earlier proposals to use the Brunswikian lens paradigm to study the emotion communication process, as it combines both the expression and perception/inference processes in a comprehensive dynamic model of emotion communication to overcome the shortcomings of focusing on only one of the component processes. The current study was designed to demonstrate the utility of the TEEP model in the domain of facial expression research. In addition to advocating the use of a comprehensive communication process approach for the research design, we propose to directly address the issue of the mechanisms involved in the process, by using the Component Process Model (CPM) of emotion (see Scherer, 1984, 2001, 2009) as a theoretical framework.

**Figure 2:**
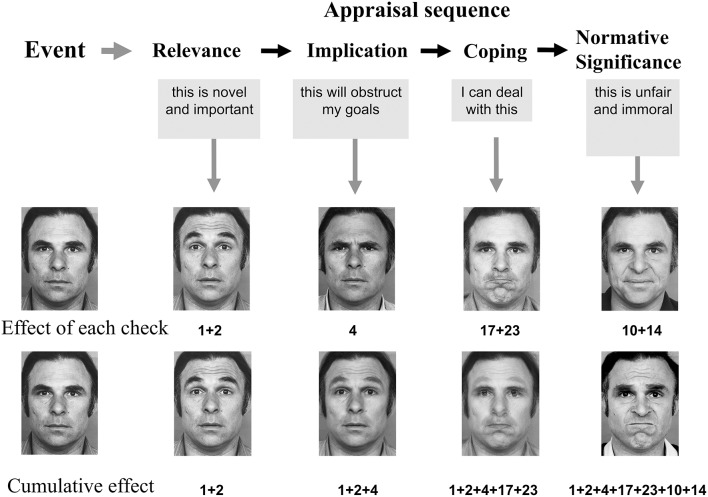
Cumulative sequential appraisal patterning as part of the Component Process Model (Scherer, 2001, 2009, 2013b). Cumulative effects were generated by additive morphing of the action unit specific photos. Adapted from Figure 19.1 in Scherer et al. (2017) (reproduced with permission from Oxford University Press).

The central assumption made by the CPM is that emotion episodes are triggered by appraisal (which can occur at multiple levels of cognitive processing, from automatic template matching to complex analytic reasoning) of events, situations, and behaviors (by oneself and others) that are of central significance for an organism's well-being, given their potential consequences and the resulting need to urgently react to the situation. The CPM assumes a sequential-cumulative mechanism, suggesting a dynamic process according to which appraisal criteria are evaluated one after another (sequence of appraisal checks) in that each subsequent check builds on the outcome of the preceding check and further differentiates and elaborates on the meaning and significance of the event for the organism and the potential response options. The most important appraisal criteria are novelty, intrinsic un/pleasantness, goal conducive/obstructiveness, control/power/coping potential, urgency of action and social or moral acceptability. The cumulative outcome of this sequential appraisal process is expected to determine the specific nature of the resulting emotion episode. During this process, the result of each appraisal check will cause efferent effects on the preparation of action tendencies (including physiological and motor-expressive responses), which accounts for the dynamic nature of the unfolding emotion episode (see Scherer, 2001, 2009, 2013b). Thus, the central assumption of the CPM is that the results of each individual appraisal check sequentially drive the dynamics and configuration of the facial expression of emotion (see Figure 2). Consequently, the sequence and pattern of movements of the facial musculature allow direct diagnosis of the underlying appraisal process and the resulting nature of the emotion episode (see Scherer, 1992; Scherer and Ellgring, 2007; Scherer et al., 2013), for further details and for similar approaches (de Melo et al., 2014; van Doorn et al., 2015).

**Figure 3:**
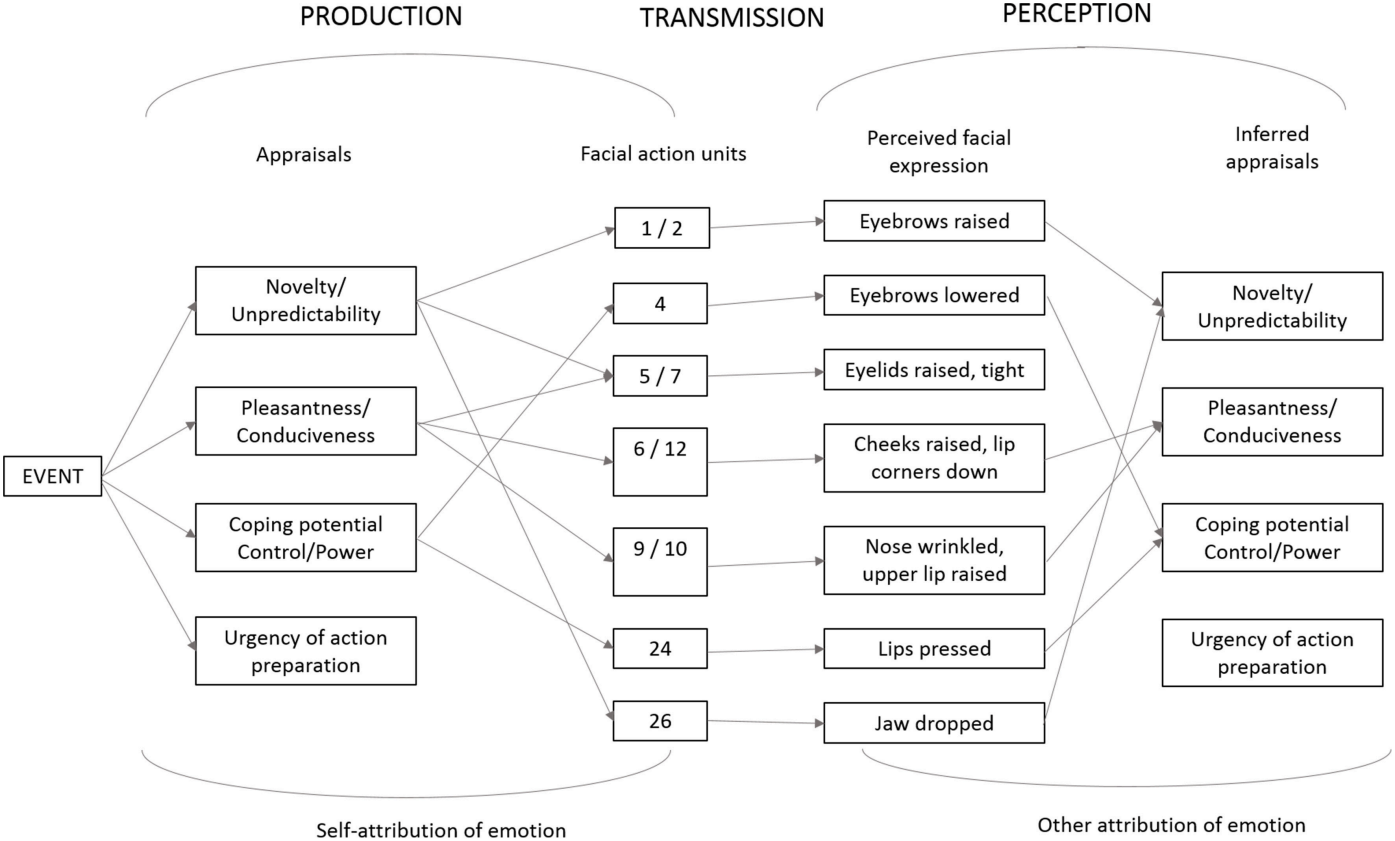
Adaptation of the TEEP model to the domain of facial expression and inference illustrating selected predictions of the Component Process Model (CPM) and empirical results. Adapted from Scherer (2013a, Figure 5.5) and Scherer et al. (2018, Figure 2).

Specific predictions for facial expression were elaborated based on several classes of determinants: (a) the effects of typical physiological changes, (b) the preparation of specific instrumental motor actions such as searching for information or approach/avoidance behaviors, and (c) the production of signals to communicate with conspecifics (see Scherer, 1984, 1992, 2001; Lee et al., 2013). As the muscles in the face and vocal tract serve many different functions in particular situations, such predictions can serve only as approximate guidelines. An illustrative example for facial movements predicted to be triggered in the sequential order of the outcomes of individual appraisal checks in fear situations is shown in Table 1. The complete set of CPM predictions (following several revisions, described in Kaiser and Wehrle (2001), Scherer and Ellgring (2007), Scherer et al. (2013), and Sergi et al. (2016) as well as the pertinent empirical evidence is provided in Scherer et al. (2018), in particular Table S1 and Appendix. Figure 3 shows an adaptation of the TEEP model described above to the facial expression domain, illustrating selected predictions of the CPM and empirical results. It should be noted that this is an example of the presumed mechanism and that the one-to-one mapping shown in the figure cannot be expected to hold in all cases.

**Table 1:** Illustration of CPM Facial Action Unit (AU) predictions for fear (Adapted from Table 1 in Scherer et al., 2018).

**Cumulative sequence of appraisal**	**Appraisal checks**	**CPM predictions for AUs generated by specific appraisal results**	**Appraisal results predicted for fear**	**AUs predicted to be produced by individual appraisal result**
1	**Novelty**			
	Sudden/unpredictable	1, 2, 4, 5, 7, 26, 38	Very high	1, 2, 4, 5, 7, 26, 38
	Familiar/predictable	–	Not applicable	
2	**Intrinsic pleasantness**			
	Pleasant	5, 26, 38 or 12, 25	Open	
	Unpleasant	4, 7, 9, 10, 15, 17, 24, 39 or 16, 19, 25, 26	Open	
3	**Goal/need significance**			
	Conduciveness	12, 25	Not applicable	
	Obstructiveness	4, 7, 23, 17	Very high	4, 7, 23, 17
4	**Coping potential**			
	High power/control	4, 5 (or 7), 23, 25 (or 23, 24)	Not applicable	
	Low power/control	15, 25, 26, 41, 43 (or 1, 2, 5, 26, 20)	Very high	1, 2, 5, 15, 20, 25, 26, 41
	CPM predictions of AUs that could potentially occur for the emotion of fear as based on the accumulation of the effects of the pertinent appraisals			1, 2, 4, 5, 7, 15, 17, 20, 23, 25, 26, 38, 41, 43

*Column 1 and 2: major appraisal checks postulated by the CPM (except self/norm compatibility) and the respective alternative outcomes; column 3: the Action units (AUs) predicted as potential expressions for the respective alternative results; column 4: the degree of pertinence of the specific appraisal outcome (high or very high) for the elicitation of fear (“Open—both outcome alternatives of a check can occur); column 5: the resulting AUs (from Column 3), expected to occur in the sequence shown in column 1. AU descriptions: 1, Inner brow raiser; 2, Outer brow raiser; 4, Brow lowerer; 5, Upper lid raiser; 7, Lid tightener; 15, Lip corner depressor; 17, Chin raiser; 20, Lip stretcher; 23, Lip tightener; 25, Lips part; 26, Jaw drop; 38, Nostril Dilator; 41, Lids droop; 43, Eye closure*.

It is important to note that the appraisal dimensions of pleasantness/goal conduciveness and control/power/coping potential are likely to be major determinants of the valence and power/dominance dimensions proposed by dimensional emotion theorists (see Fontaine et al., 2013, Chapter 2). While there is no direct equivalent for the arousal dimension regularly found in studies of affective feelings, it can be reasonably argued that on this dimension, emotional feeling does not vary by quality but by response activation, probably as a function of specific appraisal configurations, in particular the appraisals of personal relevance and urgency. A large-scale investigation of the semantic profiles of emotion words in more than 25 languages all over the world (Fontaine et al., 2013) provides strong empirical evidence for this assumption and suggests the need to add novelty/predictability as a fourth dimension (directly linked to the respective appraisals) to allow adequate differentiation of the multitude of emotion descriptions. Following this lead, we investigated the role of facial behavior in emotional communication, using both categorical and dimensional approaches (Mehu and Scherer, 2015). We used a corpus of enacted emotional expressions (GEMEP; Bänziger and Scherer, 2010; Bänziger et al., 2012) in which professional actors are instructed, with the help of scenarios, to communicate a variety of emotional experiences. The results of Study 1 in Mehu and Scherer (2015) replicated earlier findings showing that only a minority of facial action units is associated with specific emotional categories. Study 2 showed that facial behavior plays a significant role both in the detection of emotions and in the judgment of their dimensional aspects, such as valence, dominance, and unpredictability. In addition, a mediation model revealed that the association between facial behavior and recognition of the signaler's emotional intentions is mediated by perceived emotion dimensions. We concluded that, from a production perspective, facial action units convey neither specific emotions nor specific emotion dimensions, but are associated with several emotions and several dimensions. From the perceiver's perspective, facial behavior facilitated both dimensional and categorical judgments, and the former mediated the effect of facial behavior on recognition accuracy. The classification of emotional expressions into discrete categories may, therefore, rely on the perception of more general dimensions such as valence, power and arousal and, presumably, the underlying appraisals that are inferred from facial movements.

The current article extends the research approach described above in the direction of emotion enactment by professional actors, using a larger number of actors from another culture and a greater number of emotions. In Study 1, we asked professional actors to facially enact a number of major emotions and conducted a detailed, dynamic analysis of the frequency of facial actions. In Study 2 we examined to what extent emotion inferences of observers can be predicted by specific AU configurations. Finally, we estimated the appraisal criteria likely to determine the enactment of different emotions (using established semantic structure profiles of major emotion terms) and examined the relationships to the AUs coded for the actor portrayals.

## Study 1—The Role of Different AUs in Enacted Facial Emotion Expressions

### Aims

In the context of emotion enactment—using a Stanislavski-like method to induce an appropriate emotional state (see Scherer and Bänziger, 2010)—we wanted to investigate to what extent actors will use the AUs predicted to signal the appraisals that are constituent of the emotion being enacted.

### Methods

#### Participants

Professional actors, 20 in total (10 males, 10 females, with an average age of 42 years, ranging from 26 to 68 years), were invited to individual recording sessions in a test studio. We recruited these actors from the Munich Artist's Employment Agency, and each received an honorarium in accordance with professional standards. The Ethics committee of the Faculty of Psychology of the University of Geneva approved the study.

#### Design and Stimulus Preparation

The following 13 emotions were selected to be enacted: Surprise, Fear, Anger, Disgust, Contempt, Sadness, Boredom, Relief, Interest, Enjoyment, Happiness, Pride, and Amusement. Each emotion word was illustrated by a typical eliciting situation, chosen from examples in the literature, appropriate for the daily experiences of the actors. Here is an example for pride: “A hard-to-please critic praises my outstanding performance and my interpretation of a difficult part in his review of the play for a renowned newspaper.” Actors were instructed to imagine as vividly as possible that such an event happened to them and to attempt to actually feel the respective emotion and produce a realistic facial expression. To increase the ecological validity of the enactment, we asked the actors to simulate short, involuntary emotion outbreaks or affect bursts as occurring in real life (see Scherer, 1994), accompanied by a non-verbal vocalization—in this case /aah/.

#### Procedure

In the course of individual recording sessions, the actors were asked to perform the enacting of emotional expressions while being seated in front of a video camera. Six high power MultiLED softbox lights were set up to evenly distribute light over the actors' faces for best visibility of detailed facial activity^1^.

Each recording session involved two experimenters. A certified coder and experienced expert in FACS (cf. Ekman et al., 2002) served as “face experimenter.” He gave instructions to the actors and directed the “technical experimenter” who operated the camera.

The performing actor and face experimenter together read the scenario (the face experimenter aloud), before the actor gave an “ok” to signal readiness to facially express his or her emotional enactment.

#### Coding

To annotate the recordings with respect to the AUs shown by the actors, we recruited fifteen certified Facial Action Coding System (FACS, Ekman and Friesen, 1978) coders. To evaluate their performance, they were first given a subset of the recordings. For that purpose, the coders were divided into five groups of three coders each. All three coders in one group received eight recordings of one actor. Performance evaluation was based on coding speed and inter-coder agreement. Following the procedure proposed in the FACS manual, we first computed inter-coder agreement for each video for each coder with the other two coders who received the same set of videos. We then averaged these two values to get a single value for each coder. The agreement was calculated in terms of presence/absence of the Action Units within the coding for each target video. We did not compute agreement in terms of dynamics of the AUs (which is very hard to achieve; Sayette et al., 2001) nor in terms of intensities. Importantly, neither the dynamics nor the intensities were used in any of our analyses.

We excluded three coders because their average inter-coder reliabilities with the two other coders of their group were below 0.60. One more coder dropped out for private reasons. The reliabilities of the remaining 11 coders ranged from 0.65 to 0.87 (average = 0.75). The emotion enactment recordings were distributed among these 11 FACS coders. Each video was annotated by one coder.

Coders received a base payment of €15.00 per coding-hour, plus a bonus contingent on coding experience and their inter-coder reliability. On average, this amounted to an hourly payment of €18.00.

Coding instructions followed the FACS manual (Ekman et al., 2002; see also Cohn et al., 2007). Facial activity was coded in detail with regard to each occurrence of an AU, identifying onset, apex and offset with respect to both duration and intensity. For our current data analysis, we used occurrences and durations (between onset and offset) of single AUs. Different AUs appearing in sequence within an action unit combination were analyzed in accordance to predictions from the dynamic appraisal model. In addition to occurrence and intensity, potential asymmetry of each AU as well as a number of action descriptions (ADs, e.g., head and eye movements) were scored. To increase reliability three levels of intensity (1, 2, 3) were used instead of five, as suggested by Sayette et al. (2001), and applied successfully in several previous studies (e.g., Mortillaro et al., 2011; Mehu et al., 2012).

## Results

The aim of the analyses was to determine the extent to which specific AUs are used to portray specific emotions and if these correspond to the AUs that are predicted to occur (see Scherer and Ellgring, 2007; Scherer et al., 2018, and **Table 4** below) for the appraisals that are predicted as constituents for the respective emotions. While coders had scored all of the FACS categories (a total of 57 codes), we restricted the detailed analyses (i.e., those listed in the tables) to action units (AUs) from AU1 to AU28 (see the Appendix for detailed illustrated descriptions) as there are only very few predictions for action descriptors (ADs). The ADs (e.g., head raising or lowering) differ from AUs in that the authors of FACS have not specified the muscular basis for the action and have not distinguished specific behaviors as precisely as they have for the AUs. In a few cases, where there are interesting findings, the statistical coefficients for ADs are included in the text. In addition, we did not analyze AUs 25 and 26 (two degrees of mouth opening) as all actors were instructed to produce an /aah/vocalization during the emotion enactment, resulting in a ubiquitous occurrence of these two AUs directly involved in vocalization.

The dynamic frame-by-frame coding allows obtaining an indication of the approximate length of the display of particular action units during a brief affect burst. Table 2 provides a descriptive overview of the frequency of occurrence and the mean duration of different AUs for different emotions (including AUs 25 and 26, for the sake of comparison). Specifically, Table 2A contains the percentage of actors who use a specific AU to express different emotions, showing that actors vary with respect to the AUs they employ to express the different emotions. Only AUs 1, 2, 4, 6, 7, and 12 are regularly used by a larger percentage of actors. Table 2B lists the overall percentage of frames of the 78,398 frames coded in total in which the different AUs occur (column 1) and of the relative amount of time (in seconds) during which the different AUs were shown for particular emotions (the average duration across actors; columns 2–15). The table shows that average durations of AUs can vary widely, and that they are often produced for several types of emotion. AUs 1 and 2 are shown for both positive and negative emotions (possibly for greater emphasis). They are relatively brief, occurring rarely for more than 2 s. AU4 is shown for a somewhat longer period of time, mostly for negative emotions. AUs 6, 7, and 12 are primarily associated with the positive emotions, with very long durations for amusement (between 6 and 8 s) and, somewhat shorter for happiness and pride (around 3–4 s). They make briefer appearances in enactments of enjoyment and relief.

**Table 2A:** Percentage of actors displaying a particular AU when enacting a given emotion.

	**Amusement**	**Anger**	**Boredom**	**Contempt**	**Disgust**	**Enjoy**	**Fear**	**Happiness**	**Interest**	**Pride**	**Relief**	**Sadness**	**Surprise**
AU1	**30**	25	**40**	25	**30**	20	**60**	**50**	**50**	25	**30**	**55**	**40**
AU2	**40**	**30**	**35**	25	5	15	**40**	**50**	**50**	**40**	20	10	**40**
AU4	25	**45**	25	**45**	**55**	5	**70**	5	10	5	5	**75**	**40**
AU5	5	25	10	5	0	0	**50**	**40**	15	15	5	5	**40**
AU6	**100**	10	0	5	**30**	**35**	15	**80**	25	**65**	**30**	25	25
AU7	**75**	15	10	**30**	**70**	**40**	**40**	**65**	**30**	**60**	**30**	**40**	20
AU9	10	15	0	20	**50**	0	15	10	0	0	0	5	5
AU10	5	15	10	**60**	**40**	0	10	5	0	10	5	15	5
AU11	5	0	0	0	5	0	10	0	5	0	0	0	5
AU12	**100**	20	5	10	0	**70**	**30**	**90**	**55**	**95**	**75**	20	**40**
AU13	0	0	5	10	0	0	0	5	5	5	0	0	10
AU15	10	5	15	10	15	0	5	20	0	20	5	**30**	10
AU16	5	10	10	5	15	0	5	5	10	10	5	15	10
AU17	5	10	5	5	**30**	5	0	5	5	15	5	15	15
AU18	10	0	0	0	10	10	0	10	5	10	5	0	5
AU20	5	15	5	10	**35**	0	20	20	5	5	15	10	5
AU22	0	0	0	0	5	0	5	0	0	0	0	0	5
AU23	10	0	0	10	0	5	10	5	10	10	5	5	0
AU24	5	20	0	10	0	10	10	**30**	25	15	**30**	15	20
AU25	**100**	**95**	**95**	**95**	**95**	**100**	**100**	**90**	**95**	**85**	**100**	**90**	**100**
AU26	**95**	**90**	**90**	**75**	**80**	**95**	**90**	**80**	**80**	**85**	**100**	**70**	**95**
AU27	10	**35**	5	10	5	5	**35**	**45**	5	5	0	0	15
AU28	5	5	0	0	0	0	5	15	0	15	5	5	0

*Percentages >30 are bolded to provide better visibility of the major patterns*.

**Table 2B:** Occurrence and mean duration (s) of AU presence across actors.

**Occurrence in percent of frames**		**AU duration for the different emotions**													**Total duration**
**Action units**	**In percent**	**Amusement**	**Anger**	**Boredom**	**Contempt**	**Disgust**	**Enjoy**	**Fear**	**Happiness**	**Interest**	**Pride**	**Relief**	**Sadness**	**Surprise**	
AU1	12.7	0.7	0.6	**1.7**	**1.1**	**1.5**	0.6	**1.8**	**1.7**	**2.3**	0.7	**1.4**	**2.8**	**1.4**	**18.4**
AU2	9.9	1.0	**1.0**	**1.5**	**1.3**	0.3	0.3	**1.2**	**2.0**	**2.0**	1.0	0.8	0.4	**1.6**	**14.3**
AU4	12.9	0.9	**1.6**	**1.6**	**2.3**	**3.0**	0.2	**2.9**	0.1	0.4	0.0	0.4	**3.7**	**1.5**	**18.7**
AU5	3.8	0.0	0.9	0.2	0.1	0.0	0.0	**1.3**	0.7	0.3	0.3	0.1	0.3	**1.3**	**5.5**
AU6	15.3	**7.1**	0.1	0.0	0.4	**1.7**	**1.8**	0.6	**3.7**	0.8	**3.1**	**1.4**	**1.1**	0.5	**22.2**
AU7	18.5	**6.4**	0.4	0.5	**1.3**	**3.0**	**2.2**	**1.3**	**3.6**	1.0	**3.1**	**1.3**	**2.3**	0.3	**26.8**
AU9	2.5	0.5	0.3	0.0	0.5	**1.6**	0.0	0.2	0.3	0.0	0.0	0.0	0.1	0.0	**3.6**
AU10	5.1	0.5	0.5	0.3	**1.7**	**1.9**	0.0	0.5	0.2	0.0	0.5	0.2	1.0	0.2	**7.4**
AU11	1.0	0.3	0.0	0.0	0.0	0.2	0.0	0.2	0.0	0.3	0.0	0.0	0.0	0.3	**1.4**
AU12	20.9	**8.2**	0.1	0.4	0.3	0.0	**3.3**	0.7	**5.8**	**2.4**	**4.8**	**3.2**	0.4	0.8	**30.2**
AU13	0.6	0.0	0.0	0.2	0.1	0.0	0.0	0.0	0.4	0.1	0.1	0.0	0.0	0.0	0.9
AU15	3.2	0.3	0.1	0.6	0.3	0.9	0.0	0.1	0.5	0.0	0.3	0.1	**1.4**	0.2	**4.7**
AU16	1.8	0.0	0.1	0.3	0.0	0.6	0.0	0.2	0.2	0.2	0.1	0.2	0.4	0.3	**2.6**
AU17	2.3	0.1	0.2	0.1	0.0	**1.2**	0.1	0.0	0.1	0.1	0.2	0.2	0.4	0.6	**3.3**
AU18	0.4	0.1	0.0	0.0	0.0	0.1	0.1	0.0	0.2	0.0	0.1	0.0	0.0	0.0	0.6
AU20	2.3	0.1	0.4	0.0	0.1	**1.3**	0.0	0.4	0.4	0.0	0.0	0.2	0.3	0.0	**3.3**
AU22	0.3	0.0	0.0	0.0	0.0	0.2	0.0	0.2	0.0	0.0	0.0	0.0	0.0	0.0	0.4
AU23	0.4	0.1	0.0	0.0	0.1	0.0	0.0	0.2	0.0	0.1	0.0	0.0	0.0	0.0	0.6
AU24	1.6	0.0	0.3	0.0	0.1	0.0	0.0	0.2	0.4	0.4	0.4	0.2	0.1	0.1	**2.3**
AU25	38.9	**7.8**	**4.3**	**3.9**	**3.0**	**4.1**	**3.8**	**4.3**	**5.1**	**4.8**	**3.1**	**4.1**	**3.8**	**4.2**	**56.4**
AU26	29.3	**6.2**	**3.2**	**3.2**	**1.9**	**3.0**	**3.1**	**2.8**	**3.8**	**3.6**	**2.4**	**3.5**	**2.1**	**3.7**	**42.5**
AU27	2.9	0.6	1.0	0.1	0.2	0.0	0.1	0.8	0.8	0.0	0.1	0.0	0.0	0.5	**4.2**
AU28	0.6	0.2	0.1	0.0	0.0	0.0	0.0	0.1	0.4	0.0	0.1	0.0	0.1	0.0	0.9

*Column 1—Overall percentage of video frames (of the 78,398 frames coded in total) in which the different AUs occurred; Columns 2–15—relative amount of time (in seconds) during which the different AUs were shown for particular emotions (average duration across actors). Durations exceeding 1 s are bolded to provide better visibility of the major patterns*.

The dynamic frame-by-frame coding of the enactment videos allows to determine the temporal frames of AU combinations, i.e., frames in which two or more AUs are coded as being simultaneously present. As it would be impossible to study all possible combinations, we identified the most likely pairings in terms of claims in the literature. Thus, we computed new variables for the combinations AUs 6+12, AUs 1+2, AUs 1+4, and AUs 4+7. We also added AUs 6+7 given the discussion of the 2002 version of the FACS manual (see Cohn et al., 2007, p. 217). Table 2C shows the average duration per emotion for these combinations. In most cases, the simultaneous occurrence of the paired AUs is rather short—rarely exceeding 2 s.

**Table 2C:** Mean duration (s) of the simultaneous presence of major AU combinations across actors.

**Action unit combinations**	**Amusement**	**Anger**	**Boredom**	**Contempt**	**Disgust**	**Enjoy**	**Fear**	**Happiness**	**Interest**	**Pride**	**Relief**	**Sadness**	**Surprise**	**Total**
AUs 1+2	0.4	0.4	**1.3**	0.7	0.3	0.2	**1.2**	**1.7**	**2.0**	0.6	0.8	0.4	**1.3**	**11.4**
AUs 1+4	0.2	0.2	0.7	0.1	**1.4**	0.2	**1.5**	0.0	0.0	0.0	0.3	**2.3**	0.3	**7.2**
AUs 4+7	0.8	0.2	0.5	0.8	**2.1**	0.0	**1.2**	0.0	0.0	0.0	0.0	**1.2**	0.2	**7.2**
AUs 6+12	**6.9**	0.0	0.0	0.0	0.0	**1.6**	0.2	**3.5**	0.5	**2.6**	**1.3**	0.1	0.4	**17.1**
AUs 6+7	**5.9**	0.1	0.0	0.4	**1.6**	**1.4**	0.6	**3.1**	0.3	**2.3**	**1.1**	1.0	0.1	**17.7**

*Durations exceeding 1 s are bolded to provide better visibility of the major patterns*.

AUs 1+2, reflecting the orientation functions of these movements, are found in surprise, as well as, even for longer duration, in interest, happiness, and fear—all of which often have an element of novelty/unexpectedness associated with them. This element can, of course, be part of many emotions, including anger, but it probably plays a less constitutive role as in interest or fear. AUs 1+4 has the longest duration in sadness but is also found in disgust and fear. The same pattern is found for AUs 4+7, with a longer duration for disgust. AUs 6+12, but also the combination 6+7, are found for the positive emotions, in longer durations for amusement and happiness. However, 6+7 also occurs for disgust. Thus, while in some cases findings for AU combinations mirror the results for the respective individual AUs (e.g., for 6+12), in other cases (e.g., for AUs 1+4), in other cases combinations may mark rather different emotions (e.g., disgust or relief).

For the detailed statistical testing of the patterns found, we decided not to include AUs that occurred only extremely rarely, given the lack of reliability for the statistical analyses of such rare events (extremely skewed distributions). Concretely, we excluded all AUs from further analyses that occurred in <2% of the total number of frames coded (percentages ranging from 2 to 20.9%, see column 1 in Table 2B).

We calculated the number of frames during which each AU was shown in each of the 260 recordings (20 actors by 13 emotions). For each AU we computed a multivariate ANOVA with Emotion as independent variable (we did not include Actor as a factor because here we are interested in the group level rather than actor differences or actor-emotion interactions). The results allow determining whether an AU was present in a significantly greater number of frames for one emotion than the others. In all cases in which the Test of Between-Subject effects showed a significant (*p* < 0.05) effect for the Emotion factor, we computed *post-hoc* comparisons to identify homogeneous subgroups (no significant differences between members of a subgroup), and used the identification of non-overlapping subgroups (based on Waller-Duncan and Tukey-b criteria) to determine the emotions that had a high or a very high number of frames in which the respective AU occurred. Table 3 shows, for both individual AUs and AU combinations, a summary of the results for which homogeneous subgroups were identified for either or both of the *post-hoc* test criteria.

**Table 3:** Study 1—Compilation of the significant results in the multivariate ANOVA and associated *post-hoc* tests for homogeneous subgroups on the use of specific AU's for the portrayal of the 13 emotions.

**Individual action units**	**F**	**Sig**.	**Partial eta^**2**^**	**High**	**Very high**
AU4	5.581	< 0.001	0.214	con, fea, dis	sad
AU5	3.698	< 0.001	0.153	ang	fear, sur
AU6	15.429	< 0.001	0.429	pri, hap	amu
AU7	8.086	< 0.001	0.283	dis, pri, hap	amu
AU9	4.951	< 0.001	0.195		dis
AU10	2.616	0.003	0.113		con, dis
AU12	30.306	< 0.001	0.597	int, rel, enj	pri, hap, amu,
AU15	1.879	0.037	0.084		sad
AU17	2.191	0.013	0.097		dis
AU20	2.844	0.001	0.122		dis
AU27	2.448	0.005	0.107		ang
**Action unit combinations**	**F**	**Sig**.	**Partial eta**^**2**^	**High**	**Very high**
AUs 1+2	1.944	0.030	0.087		int
AUs 1+4	4.155	< 0.001	0.169	dis, fea	sad
AUs 4+7	3.202	< 0.001	0.135		dis
AUs 6+12	25.309	< 0.001	0.552	enj, pri, hap	amu
AUs 6+7	9.684	< 0.001	0.321	pri, hap	amu

*sur, Surprise; fea, Fear; ang, Anger; dis, Disgust; con, Contempt; sad, Sadness; bor, Boredom; rel, Relief; int, Interest; enj, Enjoyment; hap, Happiness; pri, Pride; amu, Amusement*.

To determine whether the pattern of AU differences found in this manner corresponds to expectations, we prepared Table 4 which shows the current results in comparison with the CPM predictions, Ekman and Friesen's (1982) EMFACS predictions, and the pattern of empirical findings reported in the literature (for details and references for the latter, see Table S1 in the Supplemental Material for Scherer et al., 2018). Only the emotions covered in all of the comparison materials are shown in Table 4. The table shows that virtually all of the individual AUs occurring with significant frequency correspond to AUs predicted by the CPM and/or EMFACS and/or have been found in earlier studies (the CPM predictions do not include head movements). It should be noted that the current results are based on highly restrictive criteria—significant main effects for overall emotion differences and significant differences with respect to non-overlapping homogeneous subgroups. Therefore, one would expect a smaller number of AUs in comparison to the predictions, which list a large set of potentially occurring AUs or the compilation of published results from rather different studies. Many of the AUs listed for certain emotions in the three rightmost columns of Table 4 were also shown for the same emotions in the current study—but they do not reach the strong criterion we set to determine the most frequently used AUs. Another reason for the relatively small number of AUs with significant emotion effects in the current study is that we requested actors, in the interest of achieving greater spontaneity, to produce the expressions in the form of very short affect bursts (together with an/aah/vocalization), which reduced the overall time span for the expression and required AUs 25 and 26 for mouth opening. In consequence, we can assume that the AUs listed in column 1 of Table 4 constitute essential elements of the facial expression of the respective emotions.

## Discussion

The results are generally in line with both the theoretical predictions and earlier empirical findings in the literature. Here we briefly review the major patterns, linking some of these to the appraisals that are considered to provide the functional basis for their production. The classic facial indicators for positive valence appraisal, AU12 (zygomaticus action, lip corners pulled up) and AU6 (cheek raiser), are present for all of the positive emotions, but we also find AUs that differentiate between them. Thus, AU7 (lid tightener) by itself and the combination AUs 6+7 are found for the expression of both pride and happiness (indicating important visual input) but not for enjoyment which is further characterized by AU43 (closing the eyes, *F* = 5.97, *p* < 0.001, eta^2^ = 0.226), a frequently observable pose for enjoyment of auditory or sensory pleasure (Mortillaro et al., 2011). For amusement we find a pattern of exaggerated length for both AUs 6 +12 and 6+7, together with AD59 (moving the head up and down, *F* = 5.19, *p* < 0.001, eta^2^ = 0.202), which probably is the byproduct of laughter. The major indicator for negative valence appraisal, AU4 (brow lowerer) is centrally involved in most negative emotions, but there are also many differentiating elements. Thus, AU10 (upper lip raiser) is found, as predicted as a result of unpleasantness appraisal, for disgust, often accompanied by AU9 (nose wrinkle) and sometimes by AU17 (raised chin) and AU20 (lip stretcher). A major indicator for unexpectedness appraisal, AU5 (upper eye lid raiser), is strongly involved in fear and anger, probably due to the scrutiny of threatening stimuli (Scherer et al., 2018). The pattern for sadness is the combination of AU1 (inner brow raiser) and AU4 (brow lowerer), together with AU15 (lip corner depression) and AD64 (eyes closed, *F* = 2.50, *p* = 0.004, eta^2^ = 0.109), suggesting low power appraisals. The facial production pattern for anger is very plausible—AUs 5, 27 (mouth stretcher) and AD57 (head forward, *F* = 2.87, *p* = 0.001, eta^2^ = 0.123): staring with the head pushed forward and mouth wide open, reminiscent of a preparation for aggression. AU4, which is generally postulated as a cue for anger as shown in the table, does not reach significance here as it is present for only short periods of time. The data for the AU combinations basically confirm the patterns found for the respective individual components, the effect sizes being rather similar. However, in some cases specific combinations attain significance although the individual components do not reach the criterion—this is notably the case for AUs 1+2 for interest and AUs 1+4 for sadness.

## Study 2—Inferences from the AUs Shown in the Emotion Portrayals

### Aims

To investigate the emotion inferences from the actor appraisals with respect to the AU configurations used by the actors, we asked judges to recognize the emotions portrayed. However, contrary to the standard emotion recognition paradigm we are not primarily interested in the accuracy of the judgments but rather in the extent to which the emotion judgments can be explained by the theoretical predictions about appraisal inferences made from specific AUs.

### Methods

#### Participants

Thirty four healthy, French-speaking subjects participated in the study (19 women, 15 men; age *M* = 24.2, *SD* = 8.7). They were recruited via announcements posted in a university building. The number of participants is sufficient to guarantee the stability of the mean ratings, which are the central dependent variables. A formal power analysis was not performed as no effect sizes based on a particular *N* were predicted.

#### Stimulus Selection and Preparation

To keep the judgment task manageable we decided to restrict the number of stimuli to be judged by using recordings for only nine of the 13 emotions portrayed, the seven listed in Table 4 (anger, fear, sadness, disgust, pride, happiness, and enjoyment) plus two (contempt and surprise). These emotions were selected based on the frequent assumption in the literature that each of them is characterized by a prototypical expression. Again, in the interest of reducing the load for the judges, we further decided to limit the number of actors to be represented. We used two criteria for the exclusion: (1) very low degree of expressivity and (2) massive presence of potential artifacts. To examine the expressivity of each actor, we summed up the durations (in terms of number of frames) of all AUs shown by her or him and computed a univariate ANOVA with actor as a factor, followed by a *post-hoc* analysis to determine which actors had significantly shorter durations for the set of AUs coded. This measure indexes both the number of different AUs shown as well as their duration. Using this information as a guide to the degree of expressivity, together with the frequency of facial mannerisms (e.g., tics, as determined by two independent expert judges), artifacts likely to affect the ratings, we excluded actors no. 1, 2, 3, 13, 18, and 21. The remaining set of 126 stimuli (9 emotions × 14 actors) plus four example videos from the same set, not included in the analysis, were used as stimuli in the judgment task.

The 126 video clips were then trimmed (by removing some seconds unrelated to emotion enactment at the beginning and end of the videos) to have roughly the same duration (between 4 and 6 s): with 1–1.5 s of neutral display, 2–3 s of emotional expression, and again 1–1.5 s of neutral display. All clips had a 1,624 × 1,080 resolution, with a 24 frames-per-second display rate. For the final version of the task, the 126 clips were arranged in one random sequence (the same for every subject), each followed by a screen (exposure duration 7 s) inviting subjects to answer. Video clips were presented without sound in order to avoid emotion judgements influenced by the “aah”-vocalizations during expressions.

#### Procedure

Three group sessions were organized on 3 different days in the same room (a computer lab) and at the same time of the day. Upon arrival, the participants were informed about the task, were reminded that, as promised on the posted announcement, the two persons with the highest scores would earn a prize, were told that they could withdraw and interrupt the study any time they wanted without penalty, and were asked to sign a written consent form. Each participant was seated in front of an individual computer and asked to read the instructions and sign the consent form. The rating instrument consisted of a digital response sheet based on Excel displayed on each participant's screen with rows corresponding to the stimulus and columns to the nine emotions. For each clip, the cell with the emotion label that in the participant's judgment best represented the facial expression seen for the respective stimulus was to be clicked. The stimuli were projected with the same resolution as their native format on a dedicated white projection surface, with an image size of 1.5 × 1.0 m. All subjects were located between 2 and 6 m from the screen, with orientation to it not exceeding 40°. Four example stimuli were used before starting to make sure everybody understood the task properly. The task then started. Halfway, a 5-min break has been made. Upon completion, after 45–50 min, participants were paid (CHF 15) and left. The two participants with the highest accuracy rate (agreement with the actor-intended emotion expression) received prizes of an additional CHF 15 each after the data analysis. The Ethics committee of the Faculty of Psychology of the University of Geneva approved the study.

## Results

The major aim of the analysis was to determine the pattern of inferences from the facial AUs shown by the actors in the emotion portrayal session.

We first used the classic approach of determining, with the help of a confusion matrix, how well the judges recognized the intended emotions and what types of confusions occurred. The confusion matrix is shown in Table 5. The raw cell entries were corrected for rater bias using the following procedure: We calculated the percentage of correct answers by dividing the number of correctly assigned labels for a given category by the overall frequency with which the respective emotion label had been used as a response by the judges. The mean percentage of accurate responses amounts to 43.7%, thus largely exceeding the chance hit rate of 11.1%. This is slightly lower than the average values for other studies on the recognition of the facial expression of emotions reported in the review by Scherer et al. (2011, Table 2). However, it should be noted that in this study a larger number of emotions (9) were to be judged compared to the usual five to six basic emotions generally used. Furthermore, actors had to respond to concrete scenarios rather than posing a predefined set of expressions resulting in variable and complex facial expressions. In addition, whereas in past research actors generally had to portray emotions with a longer utterance, here only a very brief affect bursts were to be produced. Given that the chance rate was largely exceeded and the frequent confusions (anger/contempt/disgust, fear/surprise, happiness/pride/enjoyment) are highly plausible, we can assume that the actor portrayals provide credible renderings of typical emotion expressions. This allows considering both the production results in Study 1 and the inference results reported in the next section as being representative of day-to-day emotion expressions.

**Table 4:** Study 1—Comparison of current results on AU occurrence for the portrayal of major emotions in comparison to theoretical predictions and empirical findings reported in the literature.

**Emotions**	**Current results**	**CPM predictions for specific emotions based on appraisals**	**Ekman and friesen EMFACS predictions for basic emotions**	**Empirical findings for major emotions**
Anger	5, 27, 57	4, 5, 7, 17, 23, 24, 25	4+5+7+23	4, 21, 30, 53, 57 (1, 2, 16)
Fear	4, 5, (1+4)	1, 2, 4, 5, 7, 15, 17, 20, 23, 25, 26, 38, 41, 43	1+2+4+5+7+20+26	1, 4, 5, 25, 26, 53 (2, 16)
Sadness	1, 15, 64, (1+4)	1, 2, 4, 5, 7, 15, 17, 20, 23, 25, 26, 41, 43	1+4+15	45, 53 (1, 4, 15, 17)
Disgust	4, 7, 9, 10, 17, 20, (4+7), (9+10)	4, 7, 9, 10, 15, 17, 24, 39, 16, 19, 25, 26	9+15+16	10 (4, 6, 17)
Pride	6, 7, 12 (6+7), (6+12)	4, 5, 7, 12, 23, 24, 25	–	6, 7, 10, 12, 18, 25 (1, 2, 17)
Happiness	6, 7, 12 (6+7), (6+12)	1, 2, 12, 25, 26	6+12	6, 12, 25, 53 (1, 2, 26)
Enjoyment	12, 43, (6+12)	5, 12, 25, 26, 28	–	6, 7, 10, 12, 17, 18, 25, 26, 43

*The seven rows represent seven major emotions frequently studied in the literature. Colum 1 shows the current results for these seven emotions shown in detail in Table 1. Column 2 shows the predictions of the CPM based on postulated effects of major appraisal checks for the specific emotion on the AUs that can potentially occur (head movements, AUs 50–64, were not included). Column 3 shows the EMFACS predictions proposed by Ekman and Friesen (1982). The final column shows a summary of empirical findings obtained in a number of studies that used actors to portray the emotions. AUs in parentheses were only rarely found (see Appendix Scherer et al., 2018, for details on these studies and the methods used to summarize the results). AU descriptions: 1, Inner Brow Raiser; 2, Outer Brow Raiser; 4, Brow Lowerer; 5, Upper Lid Raiser; 6, Cheek Raiser; 7, Lid Tightener; 9, Nose Wrinkler; 10, Upper Lip Raiser; 11, Nasolabial Deepener; 12, Lip Corner Puller; 13, Cheek Puffer; 14, Dimpler; 15, Lip Corner Depressor; 16, Lower Lip Depressor; 17, Chin Raiser; 18, Lip Puckerer; 20, Lip stretcher; 22, Lip Funneler; 23, Lip Tightener; 24, Lip Pressor; 25, Lips part; 26, Jaw Drop; 27, Mouth Stretch; 28, Lip Suck; 41, Lid drop; 43, Eyes Closed; 45, Blink; 53, Head up; 57, Head forward; 64, Eyes down*.

**Table 5:** Study 2—Confusion matrix for the judgments of the actor emotion portrayals (corrected for rater bias).

	**Emotion Labels Assigned by Judges**									
		**Anger**	**Fear**	**Sadness**	**Disgust**	**Pride**	**Happiness**	**Enjoyment**	**Contempt**	**Surprise**
	Anger	**50.1**	9.1	2.6	7.7	4.0	3.6	2.1	9.4	14.9
	Fear	8.4	**54.4**	3.8	6.0	1.4	1.6	2.4	2.7	14.9
	Sadness	3.1	6.4	**53.7**	10.4	3.1	3.6	1.5	9.8	4.9
Actor	Disgust	11.7	8.7	12.2	**39.2**	3.1	2.2	2.4	9.2	1.2
intention	Pride	2.3	1.9	3.4	3.2	**52.6**	23.9	12.9	8.2	5.1
	Happiness	1.6	2.5	3.4	2.8	12.0	**49.8**	9.1	3.3	14.0
	Enjoyment	2.1	3.2	4.1	2.7	15.7	11.5	**62.1**	9.6	5.4
	Contempt	15.1	4.0	8.1	16.1	3.1	1.6	3.2	**40.0**	4.7
	Surprise	5.5	9.8	8.7	11.8	4.9	2.0	4.4	8.0	**35.0**

*Percentages for accurate judgments are bolded*.

The central aim of this study was to examine the pattern of inferences judges draw from the occurrence of specific AU combinations. To identify these configurations, we ran a series of linear stepwise regressions of the complete set of AUs on each of the perceived emotion categories as dependent variables. The stepwise procedure (selecting variables to enter by smallest *p*-value of the remaining predictors at each step) determines which subset of the AUs have a significant effect on the frequency of choice of each emotion category and providing an index of the explanatory power with the help of *R*^2^. As here we are interested in the cues that are utilized to make an inference, we computed the regressions on all occurrences of the specific category in the judgment data, independently of whether it was correct (i.e., corresponding to the intended emotion) or not. For reasons of statistical stability, we again restricted the AUs to be entered into the regressions to those that occurred with a reasonable frequency (in this case mean occurrence >10%^2^) for the selected group of 14 actors and 9 emotions chosen for Study 2.

Table 6A summarizes the results for the individual AUs, providing for each inferred emotion category the predictors reaching significance (*p* < 0.05) in the final step of the regression, together with their beta weights (showing the direction and strength of the effects), as well as the adjusted R^2^ for the final equation. In the table, the AUs that correspond to the comparable patterns of the AU production in the first column of Table 4 (the summary of the MANOVA of emotion differences in the frequency of AUs shown in Study 1) are bolded (note that contempt and surprise had not been included in the comparison shown in Table 4). The results show remarkably high *R*^2^ (>0.20) scores for five of the inferred emotions suggesting that specific AUs are indeed largely responsible for the inference of underlying emotions by observers. Although the *R*^2^ values for anger, fear, disgust and contempt are lower, the results point in the same direction. Importantly, many of these configurations correspond to the theoretically predicted configurations (see columns 3–4 in Table 4). Table 6B shows the regression results for the selected AU combinations as predictors.

**Table 6:** Study 2—Linear Stepwise Regressions of (A) individual AUs and (B) AU combinations on emotion inference judgments.

	**Regressions over all 126 videos**	**Adjusted R2**	**Regressions for videos with above median percentage of correct judgment**	**Adjusted R2**
	Beta weights for AUs in the order of stepwise entry		Beta weights for AUs in the order of stepwise entry	
**(A) INDIVIDUAL AUS AS PREDICTORS**				
Anger	12 (−0.313), 1 (−0.250)	0.127	12 (−0.279)	0.060
Fear	**4 (0.248), 5 (0.226)**	0.098	**5 (0.425), 4 (0.280)**	0.256
Sadness	**1 (0.601)**, 2 (−0.412), **4 (0.145)**, 7 (0.201), 12 (−0.170)	0.437	**1 (0.688)**, 2 (−0.346), 7 (0.280), 12 (0.260), 5 (−0.244)	0.569
Disgust	12 (−0.243), 1 (−0.266), **10 (0.195**),**4 (0.205)**	0.199	12 (−0.405), 1 (−358)	0.207
Pride	**12 (0.547)**	0.324	**12 (0.316)**	0.083
Happiness	**12 (0.551)**, 27 (0.177), **6 (0.157)**	0.464	**12 (0.549)**, 27 (0.239)	0.428
Enjoyment^*^	**43 (0.561), 12 (0.252)**, 1 (−0.184)	0.437	**43 (0.646), 12 (0.365)**, 27 (−0.242)	0.566
Contempt	12 (−0.367), 5 (−0.188), 4 (−0.199)	0.114	No variables entered	
Surprise	5 (0.469)	0.219	4 (0.399), 12 (0.330)	0.136
**(B) AU COMBINATIONS AS PREDICTORS**				
Anger	6+12 (−0.253), 1+4 (−0.202)	0.070	No variables entered	
Fear	**1+4 (0.262)**	0.061	4+7 (0.332)	0.093
Sadness	**1+4 (0.626)**, 1+2 (−0.207)	0.368	**1+4 (0.640)**	0.398
Disgust	**4+7 (0.377)**, 6+12 (−0.219), 1+4 (−0.186)	0.176	No variables entered	
Pride	**6+12 (0.404)**	0.157	**6 +12 (0.331)**	0.079
Happiness	**6+12 (0.603)**, 1+2 (0.168)	0.366	**6+12 (0.641)**	0.400
Enjoyment	**6+12 (0.176)**	0.023	No variables entered	
Contempt	6+12 (−0.240), 1+4 (−0.198)	0.063	No variables entered	
Surprise	1+2 (0.337)	0.106	6+12 (0.350)	0.106

*Criteria = entry level for predictors (p < 0.05), R^2^ change ≥0.10*.

* *AU43 added as additional predictor; AUs that correspond to the comparable patterns of the AU production in the first column of Table 4 are bolded*.

Specifically, the following AUs and AU combinations for major emotions have been theoretically predicted and empirically found to frequently occur in producing specific emotions: fear—AUs 4, 5, (1+4); sadness−1, 2, 4, 7, (1+4); disgust−4, 10, (4+7); pride−12, (6+12); happiness−6, 12, (6+12); enjoyment−12, 43, (6+12). No dominant pattern is found for anger, which is not surprising given that stable predicted patterns are very rarely found in empirical expression studies. On the other hand, anger is among the best recognized emotions as shown in Table 5 (as well as in most recognition studies in the literature). One possible explanation for this apparent paradox is that, as there are many different types of anger (e.g., irritated, annoyed, offended, angry, enraged, and furious), there are many different ways to facially express (and recognize) this frequent emotion.

So far, we have only commented on the AUs with positive beta weights, that is, the presence of the respective AU is used as a marker for the inference of a specific emotion. As Table 6 shows there are also many negative beta weights, indicating that the absence *of specific other AUs* rules out the inference of the respective emotion. Given space restrictions, we cannot explore the many interesting patterns contained in these data. Note that not only accurate judgments were used in the regression; rather we used all cases in which a specific emotion was inferred for the dependent variable in the regression. This strengthens our claim that the AUs that entered the regression equation are indeed utilized as cues for the emotion inference process.

The purpose of the preceding analysis was to determine which AUs are likely to have served as cues for the inference of certain emotions, independently on whether the respective emotion intended by the actor had been correctly inferred or not. One could argue that enactments that are more correctly identified might be of particular importance to identify the AUs that are typical indicators of certain emotions. We computed the same regressions shown in Table 6 separately for those enactments that were particularly well-recognized (using only videos that were with an accuracy percentage above the median−45%). The results of this separate analysis are shown in the two rightmost columns of Tables 6A,B, allowing direct comparison. Given the reduction of the N by half requires much stronger effects in order to be entered into the regression model in the stepwise procedure. For some of the emotions, none of the AU predictors made it into the equation. However, overall we find a very similar picture and—in some cases (fear and sadness)—even higher R^2^s. We can assume that the AUs found to be predictors in both cases are indeed stable cues for the inference of certain emotions. As expected, the most stable predictors are AUs 1, 4, 6, and 12.

## Emotion Communication: Combining Expression and Inference

We have argued in the introduction that emotion inference and recognition mirror the appraisal-driven expression process as postulated by the CPM, suggesting that judges first recognize appraisal results and then categorize specific emotions based on inference rules. To directly study the relationship between the facial expressions and the appraisals that are at the origin of the emotion experience that is expressed, ideally, one has to know the actual appraisals of the person. However, for ethical and methodological reasons it is not feasible to ask for appraisal self-report during an ongoing emotional experience without substantially altering the emotion and the appraisals themselves. An alternative approach is to use the typical appraisal profiles of the target emotions. In line with the approach used in previous publications about the relationship between appraisals and facial expressions (Mortillaro et al., 2011), here we use massive empirical evidence available on the meaning of emotion terms in many different languages to determine the typical appraisals of the target emotions.

Specifically, one large-scale study (Fontaine et al., 2013) on 24 emotion terms in 28 languages identified four dimensions that are necessary to map the semantic space of emotion words: valence, power, arousal, and novelty, in this order of importance. This cross-cultural study confirmed earlier results about affective dimensions in the literature but demonstrated that valence and arousal are not sufficient to map the major emotion terms. Furthermore, the results (based on all semantic meaning facets including appraisal) provided evidence for the strong link between affective dimensions and the major appraisal checks as postulated by the CPM—(1) valence, based on pleasantness/goal conduciveness appraisal; (2) power, based on control, power, and coping potential appraisals; (3) arousal, related to appraised personal relevance and urgency of an event; and (4) novelty, based on suddenness and predictability appraisals. In a follow-up study, Gillioz et al. (2016) confirmed this finding for 80 emotion terms in the French language. The results of this study, again a four-factorial solution with valence, power, arousal and novelty, provide us with stable appraisal coordinates for the target emotion terms used in Study 2—in the form of factor scores corresponding to these terms, reproduced in Table 7. These factor scores largely confirm the theoretical predictions of the CPM (see Table 1 in Scherer, 2001, Table 5.4): for example, surprise is characterized by average values for valence, power and arousal, but high values for novelty, and happiness is characterized by positive valence, high power and arousal and medium level of novelty. We used these dimensional coordinates in the place of the emotion words used in the enactments reported in Study 1, to test whether appraisal results could be predicted only based on the facial expressions displayed by our actors.

**Table 7:** Estimated coordinates for selected French emotion words on affective dimensions [Factor scores, based on Gillioz et al. (2016)].

**Emotion**	**Valence**	**Power**	**Arousal**	**Novelty**
Anger	−1.24	1.93	0.52	−0.58
Fear	−0.38	−1.27	1.10	0.34
Sadness	−0.48	−1.31	−1.46	−0.16
Disgust	−0.79	0.02	−1.17	0.04
Pride	1.11	1.01	0.46	−1.49
Happiness	1.82	0.40	0.15	−0.20
Enjoyment	1.64	0.06	−0.15	−0.37
Contempt	−0.94	1.38	−0.91	−0.21
Surprise	0.24	−0.04	0.55	2.45

The group of judges in Study 2 attributed different emotion terms to the actor portrayal video clips (see Table 5). Based on these data, we computed a specific 4-dimensional profile for each clip by weighting the coordinates shown in Table 7 with the respective proportion of judges that inferred a specific emotion (to give greater importance to displays that allow for stable, consensual inference). We used coordinates for French emotion terms, given that our judges were speakers of French. Thus, the coordinates of the emotion words chosen by a large number of judges would be more strongly represented in the clip-specific dimensional profile.

To address the question to what extent the coordinates of the nine emotion items can be predicted by AUs, we then used these specific dimensional profiles for each clip as dependent variables in two linear stepwise regression analyses. Specifically, we regressed the AU selection used for the analyses in Study 1 to predict (a) the *expression intentions*, that is the raw coordinates for each of the four appraisal dimensions (the raw values shown in Table 7 for each emotion) and (b) the judges' inferences (the coordinates weighted by the number of judges having inferred the respective emotions). Table 8 shows the results, the left side of the table showing the regressions of the AUs on the raw coordinates reflecting the actors' enactment intention and the right side showing the regression of the AUs on the weighted coordinates for the inferred emotions.

**Table 8:** Regressions on estimated coordinates of affective dimensions for both expression (raw coordinates) and inference (weighted coordinates).

**Dimensions**	**Expression predictors (raw coordinates)**	**R2**	**Inference predictors (weighted coordinates)**	**R2**
Valence	AU12 (0.630), AU10 (−0.150), AU4 (−0.161)	0.559	AU12 (0.732), AU10 (−0.173)	0.600
Power	AU1 (−0.341), AU2 (0.273), AU4 (−0.214)	0.157	AU1 (−0.412), AU2 (0.197), AU4 (−0.290),	0.253
Arousal	AU1 (−0.187), AU5 (0.296), AU10 (−0.191), AU12 (0.232), AU27 (0.184)	0.242	AU1 (−0.274), AU2 (0.199), AU5 (0.310), AU12 (0.388), AU27 (0.216),	0.363
Novelty	AU12 (−0.320)	0.098	AU12 (−0.170)	0.093

*Criteria = entry level for predictors (p < 0.05), R^2^ change ≥0.10*.

On the expression intention side, Valence is best predicted with a very large adjusted R^2^ of 0.559. As expected, the best predictor for positive valence expression is AU12. AUs 4 and 10 predict negative valence (as one would expect from their predominance in disgust expressions). Power is not very well-predicted with an R^2^ of only 0.157. Only AU2 seems to imply high power, and AUs 1 and 4 low power. Arousal also shows a relatively low R^2^ = 0.242, with AUs 12, 5, and 27 implying high arousal, AUs 10 and 1 low arousal. The novelty dimension is the least well-predicted (R^2^ = 0.098) with AU12 for low novelty.

On the inference side, valence is again best predicted with a very large adjusted R^2^ of 0.600. As expected, the best predictor for positive valence inference is AU12. AU10 predicts negative valence inference. Power inference has a slightly higher prediction success on the inference side with an R^2^ of 0.253. Again, only AU2 signals high power, and AUs 1 and 4 low power. For inference, arousal also shows a somewhat higher *R*^2^ = 0.363, with AUs 12, 5, 27, and 2 leading to the inference of high arousal, AU1 to low arousal. As for expression intention, novelty is least well-predicted (*R*^2^ = 0.093) with AU12 for low novelty.

The main outcome of this analysis is the very high degree of equivalence in the respective AU patterns on both the expression and inference sides, which explains the accuracy results shown in Table 5. The low prediction success for power suggests that the face may not be a primary channel to communicate control, power, or coping potential, contrary to the voice (see Goudbeek and Scherer, 2010). For novelty, the low proportion of variance explained is most likely due to the low variability in novelty for the emotions studied here with the exception of surprise, and some degree, pride (as shown in Table 7). The respective predictor, AU12 for pride corresponds very well with the production side.

## Discussion and Conclusion

It should be noted that the TEEP model that served as the theoretical framework for our empirical studies, represents a structural account of the emotion communication architecture and processes. It does not specify the detailed mechanisms, on neither the expression nor perception/inference side. It remains for further theoretical and empirical work to address exactly what mechanisms are operative on the neuromotor and neurosensory levels. Thus, with respect to inference, the model does not predict whether this happens in the form of classical perception mechanisms involving templates or discrete cue combinations and (more or less conscious) inference rules, or whether the process works in an embodied fashion with the observer covertly mimicking the observed movement to derive an understanding (see Hess and Fischer, 2016). In both cases, correct communication relies on the nature of the AUs produced in expression that are objectively measurable and that serve as the input for perceived and embodied mimicry. The research reported here addresses only the issue of the nature of the AUs involved.

Based on the theoretical assumptions about the nature of the appraisal combinations that produce specific emotions, the CPM also predicts expression patterns for specific emotions (see column 2 in Table 4). Study 1 was designed to test these predictions in an enactment study using professional actors with very brief, affect-burst like non-verbal vocal utterances (see Scherer, 1994). This differs from earlier portrayal studies where generally longer verbal utterances are used, which may affect the facial expression due to the articulation movements around the mouth as well as involuntary prosodic signals in the eye and forehead regions. As shown in Table 4, the AUs consistently shown by the actors for certain emotions are in line with the theoretical predictions of the model.

Study 2 used the video stimuli with the enactments of major emotions by actors in a recognition design to obtain independent judgments as to the perceived or inferred emotions expressed. This approach served two purposes: (1) Obtaining evidence as to the representativeness of the enactments of specific emotions. The results show that this is indeed the case, hit rates exceeding chance level by a factor of 4–5 times and confusions being in line with similar patterns found in other studies; (2) Allowing us to investigate which cues are consistently utilized as markers for the inference of certain emotions.

This demonstration also supports the hypothesis described in the introduction (see also Mortillaro et al., 2012; Scherer et al., 2017, 2018), namely that the emotion inference and recognition process mirrors the production process. Specifically, our results suggest that observers use the facial expression to identify the nature of the underlying appraisals or dimensions and use inference rules to categorize and label the perceived emotion (in line with the semantic profiles of the emotion words; see Fontaine et al., 2013). We estimated the coordinates of the emotion terms used for the enactments in Study 1 on the four major affective dimensions valence, power, arousal, novelty (directly linked to the appraisal criteria of pleasantness/goal conduciveness, control/power, urgency of action, and suddenness/predictability) and then regressed the observed AU frequencies on these estimates. The results shown in Table 8 are consistent with the expectations generated by the production/perception mirroring hypothesis.

The approach we have chosen to obtain information on which appraisal dimensions are most likely to be inferred from certain AU configurations is somewhat unorthodox, using weighted estimates of the dimension coordinates for the expressive stimuli generated in Study 1 of this study as dependent variables, rather than direct ratings of appraisal dimensions. However, the latter approach would have the disadvantage of strong demand characteristics encouraging judges to consciously construct relationships between the facial expression and particular dimensions. Another major disadvantage with such a design is that the ratings of the valence dimension strongly affect all other dimensions with a powerful halo effects (see the strong evidence for these halos in Sergi et al. (2016) and Scherer et al. (2018). The advantage of our indirect method of examining the issue is that judges were focused on the emotions expressed and did not consider the appraisal dimensions explicitly, thus avoiding the occurrence of valence halos.

Overall, the results of the two studies presented here strongly confirm the utility and promise of further research on the mechanisms underlying the dynamic process of emotion expression and emotion inference using a unified theoretical framework. We suggest that further research be extended by including additional cues that may be relevant in the process of inferring emotions from facial cues. Recently, Calvo and Nummenmaa (2016) published a comprehensive integrative review on the perceptual and affective mechanisms in facial expression recognition. They conclude that (1) behavioral, neurophysiological, and computational measures indicate that basic expressions are reliably recognized and discriminated from one another, (2) affective content along the dimensions of valence and arousal is extracted early from facial expressions (but play a minimal role for categorical recognition), and (3) morphological structure of facial configurations and the visual saliency of distinctive facial cues contribute significantly to expression recognition. It seems promising to examine the interaction of such cues with the classic facial action units typically used in this research.

## Data Availability

The datasets generated for this study (coding and rating data) are available on request to the corresponding author.

## Author Contributions

KS and MM conceived the research and study 1. KS conceived study 2 and directed the research. All authors contributed to the data collection (led by AD and MU). HE and MM were responsible for facial expression production and coding. KS analyzed the data and wrote the first draft. All authors contributed in several rounds of revision.

### Conflict of Interest Statement

The authors declare that the research was conducted in the absence of any commercial or financial relationships that could be construed as a potential conflict of interest.

## Footnotes

^1^The emotion enactment was the third and final part of a series of tasks which also included first, producing facial displays of specific Action Units (AUs, according to FACS), to be used as material for automatic detection, and, second, enacting a set of scenarios with different sequences of three appraisal results (to examine sequence effects). Results of these other tasks are reported elsewhere.

^2^Note that this threshold is higher than in Study 1, as suggested by the distribution of frequencies, due to selection of more expressive actors and prototypical emotion expressions.

## Appendix

**Table:** Labels and visual illustrations of the major AUs investigated (modified from Mortillaro et al., 2011).

**AU**	**Action Name**	**Illustration**
AU1	Inner brow raise	
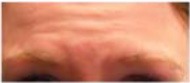
AU2	Outer brow raise	
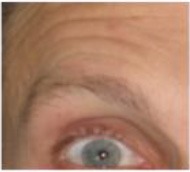
AU4	Brow lower	
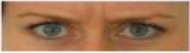
AU5	Upper lid raiser	
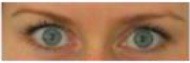
AU6	Cheek raiser	
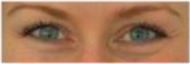
AU7	Lid tightener	
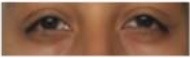
AU9	Nose wrinkler	
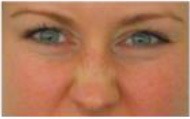
AU10	Upper lip raiser	
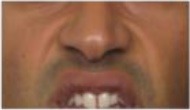
AU12	Lip corner puller	
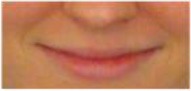
AU15	Lower corner depressor	
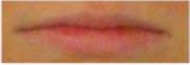
AU16	Lower lip depressor	
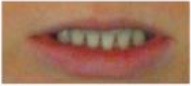
AU17	Chin raiser	
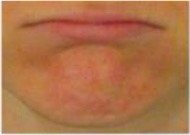
AU18	Lip puckerer	
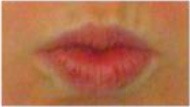
AU20	Lip stretcher	
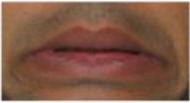
AU22	Lip funneler	
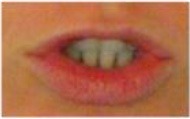
AU23	Lip tightener	
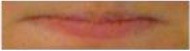
AU24	Lip pressor	
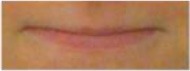
AU25	Lips part	
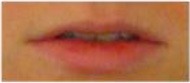
AU26	Jaw drops	
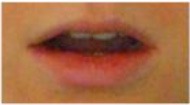
AU28	Lips suck	
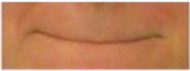
AU43	Eye closure	
